# Glucocorticoid receptor is involved in the breed-dependent transcriptional regulation of mtDNA- and nuclear-encoded mitochondria genes in the liver of newborn piglets

**DOI:** 10.1186/1746-6148-9-87

**Published:** 2013-04-26

**Authors:** Runsheng Li, Huafeng Zou, Yimin Jia, Ruqian Zhao

**Affiliations:** 1 Key Laboratory of Animal Physiology & Biochemistry, Ministry of Agriculture, Nanjing Agricultural University, Nanjing, Jiangsu, China

**Keywords:** Glucocorticoid receptor, Transcriptional regulation, Mitochondrial DNA (mtDNA), Nuclear-encoded mitochondrial genes, Liver, Piglets

## Abstract

**Background:**

Mitochondria, which are essential for the functionality of eukaryotic cells, are particularly important in metabolically active tissues such as liver. Different breeds of pigs demonstrate distinct metabolic profiles in the liver, yet little is known whether the expression and transcriptional regulation of mitochondrial genes differ between breeds.

**Results:**

Here we used male newborn Large White (LW) and Erhualian (EHL) piglets to delineate the difference in hepatic mitochondrial gene regulation between breeds. The hepatic content of ATP was significantly higher (p < 0.01) in EHL piglets, which was associated with lower mtDNA copy number (p < 0.05). Most of the mtDNA-encoded genes (10 of 13), however, were more abundantly expressed in EHL compared to LW piglets. We also detected 3 differentially expressed nuclear-encoded mitochondrial genes, among which isocitrate dehydrogenase 2 (*IDH2*) and ATP synthase, H^+^ transporting, mitochondrial Fo complex, subunit d (*ATP5H*) were expressed significantly lower, while adenylate kinase 1 (*AK1*) was significantly over expressed in EHL piglets. Compared to LW, the over expression of mtDNA-encoded genes in EHL was associated with significantly higher (p < 0.01) glucocorticoid receptor (GR) binding to the control region of mtDNA with no alterations in the methylation status. For nuclear-encoded genes, however, a negative correlation was observed between GR binding and mRNA expression of *AK1* and *ATP5H*. Moreover, higher expression of *AK1* in EHL piglets was also associated with lower cytosine methylation (p < 0.05) and hydroxymethylation (p < 0.05). In the promoter region.

**Conclusions:**

These results indicate a role of the GR in the breed-dependent regulation of mitochondrial genes in the liver of newborn piglets.

## Background

Different breeds of pigs demonstrate diverse phenotypic traits and thus serve as valuable models for animal genetics research. Erhualian (EHL) is a Chinese indigenous pig breed, being known for its early sexual maturity, large litter size, and high tolerance to roughage and stress, but its contribution to the pork industry is restricted by the slow growth rate and high adiposity [1]. In contrast, Large White (LW) pigs are selected for commercial interests such as growth rate and lean meat production. Numerous studies have been undertaken to reveal the genetic and physiological mechanisms underlying the striking breed differences [2,3]. The disparity in the regulation of energy homeostasis appears to be the main attribute for the various breed-specific traits [4,5].

Mitochondria play a critical role in energy homeostasis through regulating cellular fuel oxidation and ATP production [6]. Liver is one of the most metabolically active organs and serves as the centre of metabolism for all the major nutrients, including carbohydrates, lipids and proteins. Hepatocytes contain a large number of mitochondria which play an important role in the energy homeostasis [7]. We identified, by comparing the hepatic transcriptome of LW and EHL piglets, that mitochondrial related genes consist of 5% (48/808) of all the differentially expressed genes [5]. Among these mitochondrial related genes, isocitrate dehydrogenase 2 (*IDH2*), adenylate kinase 1 (*AK1*) and ATP synthase, H^+^ transporting, mitochondrial Fo complex, subunit d (*ATP5H*) are nuclear-encoded and closely related to cellular ATP content.

In mammals, 13 oxidative phosphorylation (OXPHOS) related proteins are encoded by mitochondria’s own genome (mtDNA). The mtDNA-encoded genes, however, usually lack 3′UTR sequences, and thus are absent in the dataset of microarray which only contains probe sets generated from 3′UTR sequences [GSE33523]. It remains to be clarified whether hepatic mtDNA-encoded genes are expressed differently between the two pig breeds.

Glucocorticoid (cortisol in pigs) is known to play critical roles in energy homeostasis [8]. Intriguingly, EHL pigs have 2–3 fold higher blood cortisol level compared to LW pigs, and glucocorticoid receptor (GR*, NR3C1*) is expressed in breed-specific patterns in hippocampus [9], muscle [10], fat [11], and liver [12]. It has been shown that the GR, besides its classic role as a nuclear receptor, can be imported into mitochondria and binds to the glucocorticoid response elements (GREs) on the control region of mtDNA [13,14]. Nevertheless, the role of GR in the regulation of nuclear- and mtDNA-encoded mitochondrial genes in different breeds of pigs remains elusive.

The overall level of DNA methylation in the liver is reported to differ between LW and EHL piglets [15]. The role of DNA methylation in transcriptional regulation has been well demonstrated in nuclear-encoded genes, including mitochondria related genes [16]. Recently, DNA methyltransferase 1 is found to translocate to the mitochondria and both 5-methylcytosine (5mC) and 5-hydroxymethylcytosine (5hmC) are reported present in mammalian mtDNA [17]. It is unclear, however, whether cytosine methylation and hydroxymethylation status on the promoter of the nuclear- or the control region of the mtDNA-encoded mitochondrial genes in the liver would differ between LW and EHL piglets.

Therefore, we have used male newborn piglets of LW and EHL to delineate the difference in hepatic mitochondria gene expression between pig breeds. The hepatic ATP content and mitochondrial copy numbers were detected, together with the expression of all the 13 mtDNA-encoded genes and the 3 differentially expressed nuclear-encoded mitochondrial genes. The impact of GR binding, DNA methylation and hydroxymethylation on these genes were evaluated by ChIP (chromatin immunoprecipitation) and MeDIP (methylated DNA immunoprecipitation) analyses.

## Methods

### Animal sampling

The newborn piglets were obtained from two neighboring pig breeding farms and sacrificed immediately after birth by exsanguination. The experiment protocol was approved by the Animal Ethics Committee at Nanjing Agricultural University, China. The slaughter and sampling procedures complied with the “Guidelines on Ethical Treatment of Experimental Animals” (2006) No. 398 set by the Ministry of Science and Technology, China and “the Regulation regarding the Management and Treatment of Experimental Animals” (2008) No.45 set by the Jiangsu Provincial People’s Government. Six newborn male piglets from three litters (2 from each litter) of each purebred EHL and LW sows were sacrificed. Liver samples were immersed in liquid nitrogen immediately after collection and then stored at −70°C.

### Hepatic ATP content and relative mtDNA copy number detection

The hepatic ATP content was measured with ENLITEN® ATP Assay System (FF2000, Promega) following the manufacturer’s instructions.

Total genomic DNA was isolated from liver samples and the relative mtDNA copy number was determined using real-time PCR as previously described with some modifications [18]. Real-time PCR was performed in Mx3000P (Stratagene, USA) with SYBR® Premix Ex Taq™ II (TaKaRa, Japan) according to the manufacturer’s instructions. Two microliter of diluted genomic DNA (10 ng/μl) was used, together with 1 μl of forward primer (10 μM) and reverse primer (10 μM), the total volume of PCR was 25 μl. Primers specific for the control region of mitochondrial DNA were used for the quantification of the mtDNA molecules, whereas primers specific for the promoter region of a nuclear gene ACTB were used for standardization (Table 1). The specificity of amplification was determined by dissociation curve analysis and PCR product sequencing. Relative mtDNA copy number was calculated with the 2^-ΔΔCt^ method [19].

**Table 1:** Primer sequences

**Primer name**	**Sequence**	**Source sequence**	**Tm (°C)**	**Used for**
*COX1*	F: TGGTGCCTGAGCAGGAATAGTG	ENSSSCG00000018075	64	mRNA quantification
	R: ATCATCGCCAAGTAGGGTTCCG			
*COX2*	F: GCTTCCAAGACGCCACTTCAC	ENSSSCG00000018078	64	mRNA quantification
	R: TGGGCATCCATTGTGCTAGTGT			
*COX3*	F: GGCTACAGGGTTTCACGGGTTG	ENSSSCG00000018082	64	mRNA quantification
	R: TCAGTATCAGGCTGCGGCTTCA			
*ND3*	F: AGCACGCCTCCCATTCTCAAT	ENSSSCG00000018084	64	mRNA quantification
	R: TGCTAGGCTTGCTGCTAGTAGG			
*CYTB*	F: CTGAGGAGCTACGGTCATCACA	ENSSSCG00000018094	64	mRNA quantification
	R: GCTGCGAGGGCGGTAATGAT			
*ND1*	F: TCCTACTGGCCGTAGCATTCCT	ENSSSCG00000018065	64	mRNA quantification
	R: TTGAGGATGTGGCTGGTCGTAG			
*ND2*	F: ATCGGAGGGTGAGGAGGGCTAA	ENSSSCG00000018069	64	mRNA quantification
	R: GTTGTGGTTGCTGAGCTGTGGA			
*ND4L*	F: GATCGCCCTTGCAGGGTTACTT	ENSSSCG00000018086	64	mRNA quantification
	R: CTAGTGCAGCTTCGCAGGCT			
*ND4*	F: TCGCCTATTCATCAGTAAGTCA	ENSSSCG00000018087	64	mRNA quantification
	R: GGATTATGGTTCGGCTGTGTA			
*ND5*	F: CGGATGAGAAGGCGTAGGAA	ENSSSCG00000018091	64	mRNA quantification
	R: GCGGTTGTATAGGATTGCTTGT			
*ND6*	F: ACTGCTATGGCTACTGAGATGT	ENSSSCG00000018092	64	mRNA quantification
	R: CTTCCTCTTCCTTCAACGCATA			
*ATP6*	F: ACTCATTCACACCCACCACACA	ENSSSCG00000018081	64	mRNA quantification
	R: CCTGCTGTAATGTTGGCTGTCA			
*ATP8*	F: TGCCACAACTAGATACATCC	ENSSSCG00000018080	62	mRNA quantification
	R: GCTTGCTGGGTATGAGTAG			
*IDH2*	F: GGACAGTCACCCGCCACTA	ENSSSCG00000001852	62	mRNA quantification
	R: CGTCCAGGCAAAGATGCTG			
*AK1*	F: TTGGACATGCTCCGAGACGC	ENSSSCG00000005627	62	mRNA quantification
	R: CCGATCTTCCGCTCAAACTCTT			
*ATP5H*	F: CTACCTGAGAAGCCACCTGC	NM_001244684	62	mRNA quantification
	R: GCTGCCCACCTATGACCAC			
*PPIA*	F: GACTGAGTGGTTGGATGG	ENSSSCG00000016737	62	mRNA quantification
	R: TGATCTTCTTGCTGGTCTT			
*18S*	F: CCCACGGAATCGAGAAAGAG	ENSSSCG00000001502	64	DNA contamination detection
	R: TTGACGGAAGGGCACCA			
MT_controlregion	F: CCCTATAACGCCTTGCCAAACC		62	ChIP, MeDIP, mtDNA copy number
	R: GGGTAGGTGCCTGCTTTCGTAG			
MT_GRE_neg	F: TCGCCTATTCATCAGTAAGT		62	ChIP
	R: GAGGATGTTAGTCCGTGGG			
MT_MeDIP_neg	F: CTCAGTAGCCATAGCAGTA		62	MeDIP
	R: TGGACTTGGGTTGATTGT			
*IDH2*_promoter	F: GGTTCAACAAGCCTTACTCACA		62	ChIP, MeDIP
	R: GGAGACAGGAGCCGCATAG			
*AK1*_promoter	F: TGGGAGATGGATATGTGGGC		62	ChIP, MeDIP
	R: GCATGGATTCTGGGGACTGT			
*ATP5H*_promoter	F: CCAAAGGTACATGGACTGC		62	ChIP, MeDIP
	R: GCATCTTGGGTGGTTTTAT			
*PPIA*_GRE_neg	F: GTTCACAGGGTGGTGACTT		62	ChIP
	R: CAGGACCCGTATGCTTCA			
*ACTB*_MeDIP_neg	F: CTGGGCATCAGAACCTGT		62	MeDIP, mtDNA copy number
	R: GAGCAATCCCCTGAAGAA			

### RNA isolation and mRNA quantification

Total RNA was isolated from liver using the Trizol reagent (Invitrogen, USA), according to the manufacturer’s instructions. Concentration of the extracted RNA was measured using NanoDrop ND-1000 Spectrophotometer [20]. RNA integrity was confirmed by denaturing agarose electrophoresis. To assess the DNA contamination, the real-time PCR was carried out using 2 μl diluted RNA (about 1 ng/μl) as template with the primers of 18S. Samples with Ct values larger than 35 were consider not contaminated by genomic DNA. M-MLV reverse transcriptase (Promega, USA) and dN6 random primer (Takara, Japan) were used to synthesize cDNA from 2 μg of total RNA from each sample according to the manufacturer’s instructions, the final cDNA volume of reverse transcription was 25 μl. After that, 2 μl of diluted cDNA (1:100) was used for the real-time PCR detection. Several reference genes (*18S*, *PLC4*, *GAPDH*, *ACTB* and *PPIA*) were tested for mRNA quantification. The expression stability was evaluated by geNorm [21] and peptidylprolyl isomerase A (*PPIA*) was shown to be most the stable. Additionally, *PPIA* seems to be stable between indigenous Chinese pig breeds and western breeds [22]. Therefore *PPIA* was used as the reference gene. The real-time PCR procedure in this section was similar regarding mtDNA copy number detection, except the template. Relative mRNA expression was calculated with the 2^-ΔΔCt^ method. All primers used for this experiment were listed in Table 1.

### Chromatin immunoprecipitation (ChIP)

ChIP analysis was performed as previously described with some modifications [14,23]. Briefly, 200 mg of frozen liver samples were ground in liquid nitrogen and washed with phosphate-buffered saline (PBS) containing protease inhibitor cocktail (Roche, USA). After cross-linking in 1% formaldehyde, the reaction was stopped with 2.5 M glycine. The pellets were washed with PBS and lysed with SDS lysis buffer (50 mM Tris–HCl pH 8.0, 10 mM EDTA, 1% SDS) containing protease inhibitors. Cross-linked samples were sonicated for 10 min on ice with 10-s on/off intervals (Sonics Vibra, USA). The samples were then centrifuged at 12,000 rpm for 10 min at 4°C to remove cell debris from the crude chromatin preparations. The average length of sonicated chromatin was around 200 to 500 bp, determined by resolving it on a 1% agarose gel. After pre-clearance of the resulting chromatin with pre-cleared Protein A/G PLUS-Agarose (sc-2003, Santa Cruz), the immunoprecipitation was performed with 2 μg of a specific GR antibody (sc-1004x, Santa Cruz) overnight at 4°C. DNA fragments were released by reverse cross-linking from the immunoprecipitated complex at 65°C for 5 h. Thereafter the DNA fragments were treated with Proteinase K (Sunshine, China) at 45°C for 1 h. After phenol/chloroform extraction, the DNA fragments were precipitated by ethanol. Finally, the samples were resuspended in 100 μl Tris buffer (10 mM Tris, pH 8.5) and 2 μl of the immunoprecipitated DNA were used as a template for the real-time PCR detection.

The primers designed for the coding region of PPIA with absence of the GRE were used as the negative control for the enrichment of GR binding on the promoter of nuclear encoded genes. For mtDNA, primers designed for the mtDNA region without any putative GRE were used as the negative control. The negative control primers are listed in Table 1. The real-time PCR procedure in this section was similar to that used for mtDNA copy number detection, except the template. The relative GR binding was calculated with the 2^-ΔΔCt^ method, using the negative control primer as a reference.

### 5mC and 5hmC Immunoprecipitation

MeDIP analysis was performed as previously described [24] with some modifications. Total genome DNA was sonicated (10 min on ice with 10 s on/off intervals) to yield DNA fragments of 200 to 500 bp in length (Sonics Vibra, USA). One microgram of fragmented DNA was heat denatured to produce single-stranded DNA and immunoprecipitation was performed overnight at 4°C with 1 μg anti-5mC antibody (ab10805, Abcam), or anti-5hmC antibody (39999, Active Motif). Pre-cleared Protein A/G PLUS-Agarose (sc-2003, Santa Cruz) was used to immunoprecipitate the antibody/DNA complex. The beads bound with immune complexes were washed to remove nonspecific binding and resuspended in 250 μl of digestion buffer containing proteinase K. Finally, the MeDIP DNA was purified with phenol/chloroform extracting and then ethanol precipitated. The resulting DNA fragments were then resuspended in 100 μl Tris buffer (10 mM Tris, pH 8.5) and 2 μl of the DNA was used for the real-time PCR detection.

The primers designed for the *ACTB* promoter region without the CpG site were used as a negative control. For mtDNA, the primers designed for the mtDNA region without CpG site were used. All primers are listed in Table 1. The real-time PCR procedure in this section was similar to that used for mtDNA copy number detection except the template. The relative methylation status was calculated using the 2^-ΔΔCt^ method. The negative control primer was used as the reference.

### Bioinformatics approach and statistical analysis

The 5′ flanking sequence of all the nuclear genes investigated was fetched from Ensembl (Sscrofa10.2, Ensembl release 69). The 5000 bp of the 5′ flanking sequence of the nuclear-encoded gene was used to predict CpG islands and GREs. The control region of the mitochondrion was defined as in previous reports [14,25].

The CpG islands on the promoters of the candidate genes were assessed by Methyl Primer Express v1.0 (Applied Biosystems, USA) using the following criteria: %GC > 50%, length > 200 bp, CpG observed/CpG expected > 0.6. The putative GREs in the promoter region were predicted by Transcription Element Search System (TESS, http://www.cbil.upenn.edu/cgi-bin/tess/tess). The promoter region containing multiple GREs was used to design primers for GR binding detection. The promoter region with a high density of GREs, inside the CpG islands was used to design the primer for the GR binding and methylation status detection.

To access the correlations of ATP content, mtDNA copy number, gene expression, DNA (hydroxy)methylation status and GR binding status in all 12 samples (6 LW and 6 EHL), a cluster analysis was carried out using all previously listed characteristics. Each characteristic value was log2 transformed for the cluster analysis. The hierarchical cluster analysis was preformed with MeV software (version 4.2.6). Correlation (centred) similarity matrix and average linkage algorithms were used in the cluster analysis.

All data are presented as the mean ± SEM and were analyzed using the *t* test for independent-samples with SPSS 17.0 for windows. Differences were considered significant when p < 0.05.

## Results

### Hepatic ATP content and mtDNA copy number in newborn piglets differ between breeds

**Figure 1:**
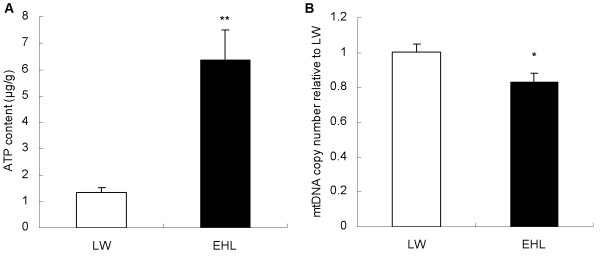
**Hepatic ATP content (A) and the relative mtDNA copy number (B) in newborn EHL and LW piglets.** Values are mean ± SEM, n = 6/breed. * means p < 0.05, ** means p < 0.01.

The hepatic ATP content was almost 5 fold higher (p < 0.01, Figure 1A) in EHL than that in LW piglets (6.3 vs. 1.3 μg/g). The copy number of mtDNA in the liver, however, was significantly lower in EHL than in LW (p < 0.05, Figure 1B).

### The expression of mtDNA-encoded genes and nuclear-encoded mitochondrial genes

**Figure 2:**
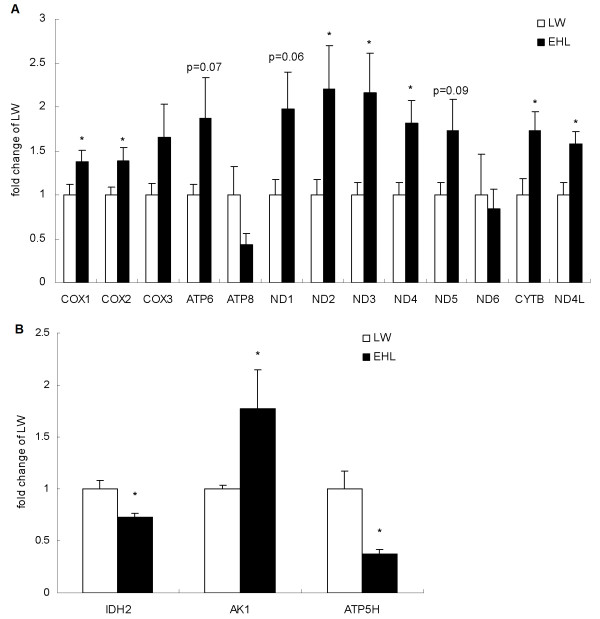
**Hepatic expression of mtDNA- (A) and nuclear-encoded (B) mitochondrial genes in EHL and LW pigs.** Values are mean ± SEM, n = 6/breed. * means p < 0.05, p values between 0.05 and 0.1 are marked above the bar.

In contrast to the mtDNA copy number, most of the mtDNA-encoded genes (10 of 13) showed a higher expression in EHL than that in LW (Figure 2A). *COX1*, *COX2*, *ND2*, *ND3*, *ND4*, *CYTB* and *ND4L* were significantly up-regulated in the liver of EHL piglets compared to LW (p < 0.05), while *ATP6*, *ND1*, *ND5* tended to be higher (p < 0.1). For the three nuclear-encoded mitochondrial genes investigated, *IDH2* and *ATP5H* were significantly down-regulated, while *AK1* was significantly up-regulated in EHL when compared to LW (Figure 2B).

### GR binding and methylation status of the control region of mtDNA

**Figure 3:**
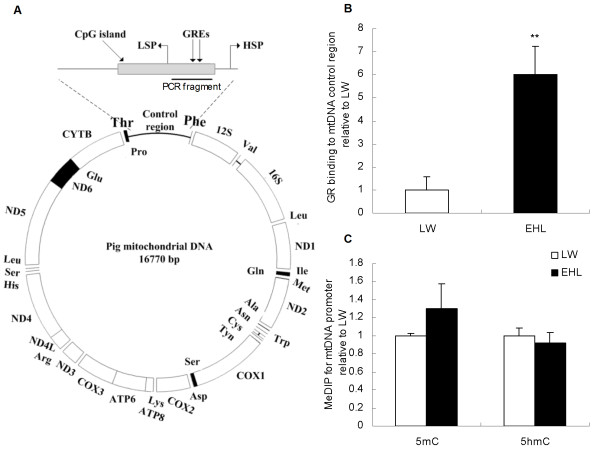
**Schematic structure of pig mtDNA (A), GR binding (B), and DNA (hydroxyl)methylation status of CpG island on the control region of mtDNA (C).** Values are mean ± SEM, n = 6/breed. ** means p < 0.01 between breeds; GREs, glucocorticoid response elements; HSP, heavy strand promoter; LSP, light strand promoter.

The control region of the mtDNA spans from 15436 to 16770 bp containing a CpG island (from 15856 to 16655 bp). Two putative GR binding sites were predicted in this region (Figure 3A). GR binding to the control region of mtDNA was significantly higher (P < 0.01) in EHL than that in LW (Figure 3B), whereas the methylation status of this region did not show any breed difference (Figure 3C).

### GR binding and methylation status of promoter of nuclear-encoded mitochondrial genes

**Figure 4:**
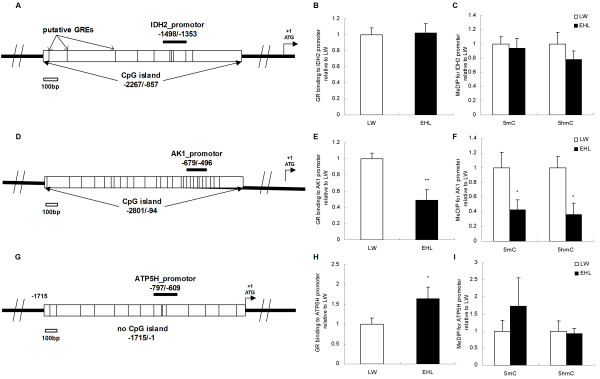
**GR binding and (hydroxyl)methylation status of the promoter of nuclear-encoded mitochondrial genes.** Values are mean ± SEM, n = 6/breed. * means p < 0.05 between breeds, ** means p < 0.01. GREs, glucocorticoid response elements. (**A**) Schematic structure of *IDH2* promoter. (**B**) GR binding to *IDH2* promoter. (**C**) DNA methylation and hydroxymethylation status of the CpG island on *IDH2* promoter. (**D**) Schematic structure of *AK1* promoter. (**E**) GR binding to *AK1* promoter. (**F**) DNA methylation and hydroxymethylation status of the CpG island on *AK1* promoter. (**G**) Schematic structure of *ATP5H* promoter. (**H**) GR binding to *ATP5H* promoter. (**I**) DNA methylation and hydroxymethylation status of *ATP5H* promoter.

Figure 4 shows the promoter regions of *IDH2* (Figure 4A), *AK1* (Figure 4D) and *ATP5H* (Figure 4G). Both *IDH2* and *AK1* have a CpG island in their promoter region. There are 10 and 29 putative GREs in the CpG island of *IDH2* and *AK1*, respectively. Since there was a sequence gap located in the proximal 5′ flanking sequence of porcine *ATP5H* gene, we used the sequence of 1715 bp prior to the ATG site for further assessment. No CpG islands were found within the sequence, but 15 putative GREs were observed.

Neither GR binding nor the methylation status of the *IDH2* promoter region showed differences between the two pig breeds (Figure 4B, C). GR binding to the *AK1* promoter region, however, was significantly lower in EHL, associated with lower levels of cytosine methylation (p < 0.05) and hydroxymethylation (p < 0.05), compared to LW piglets (Figure 4E, F). In contrast, GR binding to the promoter region of *ATP5H* was significantly higher (p < 0.05) in EHL compared to LW, while the methylation and hydroxymethylation status did not differ between breeds (Figure 4H, I).

### The hierarchical cluster analysis of all characteristics

**Figure 5:**
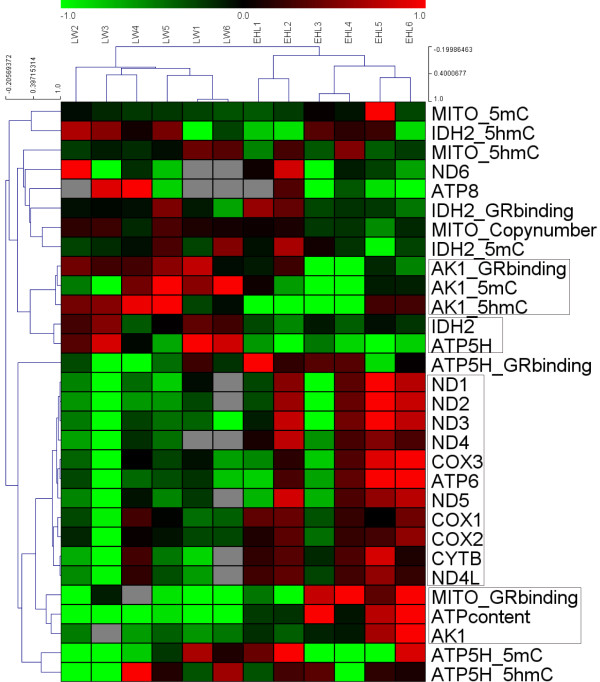
**Hierarchical cluster analysis of the characteristics in liver from Large White (LW) and Erhualian (EHL) piglets.** The figure was drawn by MeV software (version 4.2.6). Correlation (centred) similarity matrix and average linkage algorithms were used in the cluster analysis. Each row represents an individual characteristic, and each column represents a sample. The dendrogram at the left side and the top displays similarity of expression among characteristics and samples individually. The color legend represents the characteristic level, with red indicating high expression levels and green indicating low expression levels, while the gray blocks in the figure indicate missing values. The codes on the legend are log2-transformed values.

By the cluster analysis for samples, the EHL and LW piglets can be divided into two clusters. Piglets from the same sow (EHL1 and EHL 2, EHL 3 and EHL 4, EHL 5 and EHL 6, LW3 and LW4) seem to have more similar pattern. In the cluster analysis for characteristics, the 11 of 13 (except *ND6* and *ATP8*) mtDNA-encoded genes make up the largest cluster, all 11 genes showed very similar expression patterns. The ATP content was mostly related to *AK1* expression and the GR binding to the mtDNA. The expressions of *IDH2* and *ATP5H* were clustered into the same one. GR binding and (hydroxy)methylation status of the *AK1* promoter were highly correlated (Figure 5).

## Discussion

Like other mammals, the pig contains a mtDNA genome which encodes 13 proteins, along with 12 s, 16 s rRNAs and 22 tRNAs. The 13 proteins synthesized on mitochondrial ribosomes are incorporated into respiratory complex I, III, IV, and V along with approximately 67-nuclear-encoded subunits [26]. Previous studies have shown breed differences in mitochondrial function in muscle and fat in the pig [27,28]. The transcript profiling of ESTs from the semimembranosus muscle demonstrates elevated expression of mitochondrial related enzymes (NADH dehydrogenase, cytocrome c oxidase and cytocrome b reductase) in the skeletal muscle of Piau, a naturalized Brazilian pig breed with slow growth rate, high fat deposition and favorable meat quality, compared to commercial breeds (Large White and Duroc), implicating breed differences in oxidative and glycolytic metabolism [29]. Similar breed differences were reported in Korean native pigs who demonstrate higher expression of the 3 mitochondrial proteins: succinate dehydrogenase, NADH-ubiquinone oxidoreductase, and glycerol-3-phosphate dehydrogenase in the muscle as compared with Landrace [4]. In contrast, genes involved in mitochondrial energy metabolism are reported to be more abundantly expressed in adipose tissue of LW pigs, compared to Basque pigs showing low growth rate and high fat content [30].

Previous studies indicate striking breed differences in hepatic metabolism in the pig [5,31]. Although it is well known that hepatic metabolism is closely related to mitochondrial functions, no direct evidence of breed differences in mitochondrial function is available in the pig. Here we describe, for the first time to our knowledge, breed differences in mitochondrial function in porcine liver. Most of the mtDNA-encoded genes are expressed in higher abundance in the liver of EHL piglets, indicating more active OXPHOS function which results in higher deposition of hepatic ATP. Nonetheless, mRNA expression is not directly related to the function. In some cases, the levels of mRNA and protein are inversely correlated. Therefore, it is possible that some OXPHOS related genes were expressed lower in EHL piglets, while the majority of the relevant genes were more highly expressed in EHL piglets, when compared with LW. Additionally, the lower *IDH2* and *ATP5H* gene expression in EHL could be a feed-back effect of the higher ATP content. Nevertheless, more direct evidence is still needed to support our presumption. For ATP8, we didn’t observe a lower expression because of the high standard deviation in each group. Although the PCR product of ATP8 primer was confirmed by sequencing, our primer did not appear to amplify the ATP8 sequence very well. Note that there are 3 missing values of the LW group because of a bad dissociation curve (Figure 5). The expression of ND6 was also not affected by the GR. This may be explained by the ND6 being encoded by the light strain, while the other genes were encoded by the heavy strain.

The differences in mitochondrial function between breeds may contribute to some physiological properties. In our previous “Gene Ontology” analysis using hepatic differentially expressed genes between LW and EHL piglets, the “ATP binding” ranked the third in significantly enriched “molecular function” terms [5]. The higher liver ATP content in EHL may activate several transmembrane proteins that use ATP hydrolysis as an energy source for the transport of a variety of substances through cellular membranes, especially cholesterol and lipoprotein [32,33]. And when compared to LW, the EHL piglets showed elevated serum total cholesterol, high density lipoprotein cholesterol and low density lipoprotein cholesterol, as well as higher liver content of cholesterol [5]. In type 1 diabetes the liver mitochondrial proteome shifts to support ATP production [34]. The EHL piglets, which has lower serum insulin than LW, gets higher hepatic OXPHOS activity.

To our surprise, reduced mtDNA copy number was detected in the liver of the EHL piglets, indicating lower mitochondrial biogenesis. Improved hepatic mitochondrial function is usually associated with enhanced mitochondrial biogenesis, which sometimes corresponds with up-regulation of peroxisome proliferator-activated receptor γ coactivator-1α (PGC-1α, *PPARGC1A*) [35]. *PPARGC1A* was up-regulated in several liver transcriptome datasets when treated with dexamethasone [GSE24255, GSE24256 and GSE34229], but did not differ between EHL and LW piglets in our porcine liver transcriptome dataset [GSE33523]. The mtDNA copy number, however, was not always associated with mtDNA transcription and mitochondrial biogenesis. In our correlation analysis, the mtDNA copy number was even reversely related to the ATP content (data not shown). Previous research on the rat showed that an age-related decrease in mtDNA copy number was not associated with COX transcript levels in liver and red soleus muscle [36]. Increased mtDNA copy number by exogenous mitochondrial transcription factor A (*TFAM*) in mouse did not result in higher respiratory chain capacity or mitochondrial mass [37].

GR, as a transcription factor, can activate or repress the expression of nuclear-encoded genes, depending on the nature of the GREs. It is known that some motifs like “GGTACANNNTGTTCT” in the promoter region have been recognized as a positively modulated GRE [38], whereas the motif like “TGGACG” was suggested as a negatively modulated GRE (nGRE) [39,40]. The GREs predicted in the promoters of the 3 nuclear-encoded mitochondrial genes are predominantly nGREs. The density of putative GRE in the promoter region may imply a probability of the GR binding to the region. The average GRE number in the promoter region of *IDH2*, *AK1* and *ATP5H* is 0.71, 1.07 and 0.88 per 100 bp, respectively (Figure 4). High density of GREs in the promoter region of a gene indicates that this gene may be more sensitive to glucocorticoids. In this study, we found that GR binding to the promoter region is inversely related to the expression of *AK1* and *ATP5H* in LW and EHL piglets, respectively, indicating an inhibitory role of the GR in the transcriptional regulation of these two genes. Unlike nuclear encoded genes whose transcription is controlled by their respective promoters, mtDNA-encoded genes share a common control region, also known as the D-loop region [41]. Several single nucleotide polymorphisms (SNPs) have been identified in the control region of mtDNA between pig breeds [25,42]. In almost all situations, the SNPs between the pig breeds only show differences in proportion. The role of the GR in the regulation of mtDNA transcription has not been described in the pig. GRE binding sites like “CAGAGTGTACA” and “TCTTATAAAACA” were predicted in the control region of mtDNA. Glucocorticoid is reported to increase mitochondrial gene expression and OXPHOS activity by increasing the binding of GR to mtDNA [14]. In agreement with these findings, GR binding to the control region of mtDNA was 5 fold higher in the liver of EHL piglets compared to LW. By the cluster analysis, we can find that the GR binding was closely related to the expression of 11 mtDNA-encoded genes. These findings indicate complex roles of the GR in the regulation of mtDNA- and nuclear-encoded mitochondrial genes in the pig.

Although the overall level of DNA methylation in the liver was reported to be different between LW and EHL piglets [15], we failed to show breed differences in the level of cytosine methylation and hydroxymethylation in the control region of mtDNA. Among the 3 nuclear-encoded mitochondrial genes investigated, only *AK1* is differentially modified in the promoter region, including both cytosine methylation and hydroxymethylation. The function of 5hmC in the nuclear genome is not yet clear. The 5hmC sites mainly located in the coding sequence, can alter local chromatin structure or associate with the demethylation process to induce transcription [43]. Meanwhile, the 5hmC located in the promoter region but not the gene body usually negatively regulates gene expression, and functions like 5mC [44,45]. The lower levels of cytosine methylation and hydroximethylation in the promoter region of the *AK1* gene in the liver of EHL piglets could be associated with the higher expression levels of *AK1* observed in EHL piglets in comparison with LW ones. Cluster analysis showed that 5mC and 5hmC level in the promoter region of *AK1* were closely correlated, both of them were negatively correlated with the expression of *AK1* (data not shown). Further studies are needed to understand whether GR binding interacts with DNA methylation to regulate gene transcription.

## Conclusions

Our results provide the first evidence for the breed-specific regulation of hepatic mitochondrial gene expression in the pig. GR is involved in the regulation of both nuclear- and mtDNA-encoded mitochondrial genes in porcine liver. The disparity in mitochondria OXPHOS function between breeds results, at least partly, from the breed-dependent role of GR in the regulation of mtDNA- and nuclear- encoded mitochondrial genes in the liver of newborn LW and EHL piglets. Our findings shed new light on our understanding of the molecular mechanisms underlying breed disparities in the pig.

## Abbreviations

LW: Large White; EHL: Erhualian; mtDNA: mitochondrial DNA; OXPHOS: Oxidative phosphorylation; GR: Glucocorticoid receptor; GRE: Glucocorticoid response element; 5mC: 5-methylcytosine; 5hmC: 5-hydroxymethylcytosine; ChIP: Chromatin immunoprecipitation; MeDIP: Methylated DNA immunoprecipitation.

## Competing interests

The authors declare that they have no competing interests.

## Authors’ contributions

RZ designed the experiment, supervised laboratory work and critically revised the manuscript; RL mainly preformed the experiments and analyzed the data; HZ provided samples and carried out the ChIP experiment; YJ assisted with discussion of results and writing the manuscript. All authors read the manuscript and approved it in its final version.
